# Performance Assessment of a Humidity Measurement System and Its Use to Evaluate Moisture Characteristics of Wheelchair Cushions at the User–Seat Interface

**DOI:** 10.3390/s17040775

**Published:** 2017-04-05

**Authors:** Zhuofu Liu, Haifeng Cheng, Zhongming Luo, Vincenzo Cascioli, Andrew I. Heusch, Nadia R. Nair, Peter W. McCarthy

**Affiliations:** 1 The Higher Educational Key Laboratory for Measuring & Control Technology and Instrumentations of Heilongjiang Province, Harbin University of Science and Technology, Harbin 150080, China; mm7723@163.com (H.C.); 13936198757@163.com (Z.L.); 2 Murdoch University Chiropractic Clinic, Murdoch University, Murdoch 6150, Australia; v.cascioli@murdoch.edu.au; 3 Welsh Institute of Chiropractic, University of South Wales, Treforest, Pontypridd CF37 1DL, UK; aiheusch@uwclub.net (A.I.H.); nadianair@yahoo.co.uk (N.R.N.); 4 Faculty of Life Science and Education, University of South Wales, Treforest, Pontypridd CF37 1DL, UK; peter.mccarthy@southwales.ac.uk

**Keywords:** humidity measurement, cushion, wheelchair, sensor position

## Abstract

Little is known about the changes in moisture that occur at the body–seat interface during sitting. However, as increased moisture can add to the risk of skin damage, we have developed an array of MEMS (Micro-Electro-Mechanical System) humidity sensors to measure at this interface. Sensors were first evaluated against traceable standards, followed by use in a cross-over field test (*n* = 11; 20 min duration) using different wheelchair cushions (foam and gel). Relative humidity (RH) was measured at the left mid-thigh, right mid-thigh and coccyx. Sensors were shown to be unaffected by loading and showed highly reliable responses to measured changes in humidity, varying little from the traceable standard (<5%). Field-test data, smoothed through a moving average filter, revealed significant differences between the three chosen locations and between the gel and foam cushions. Maximum RH was attained in less than five minutes regardless of cushion material (foam or gel). Importantly, RH does not appear to distribute uniformly over the body–seat interface; suggesting multiple sensor positions would appear essential for effectively monitoring moisture in this interface. Material properties of the cushions appear to have a significant effect on RH characteristics (profile) at the body–seat interface, but not necessarily the time to peak moisture.

## 1. Introduction

Among the sedentary life-style related chronic diseases, epidermal ulceration is one of the most painful, hardest to treat successfully and has the potential to create life-threatening complications [1]. This situation is made more serious for those wheelchair users who cannot reposition themselves because of their incapacity, inability or simply because they may not have sufficient sensory awareness to recognise when their skin integrity is threatened due to their immobility. The local skin and subcutaneous tissue blood supply (including muscle) can become severely compromised without regular interventions that target relieving the mechanical stress and ventilating the microenvironment at the body–seat interface. The consequence of insufficient or inefficient intervention will initially be deterioration of the epithelial and subcutaneous tissues, increasing friability of the tissue and eventual tissue necrosis; resulting in an open wound (ulcer) [1,2].

Treatment of skin ulcers is costly [3,4] and rarely either easy or successful [5]. As a consequence, it is essential to understand more about the body–seat interface environment in order to establish strategies capable of preventing skin ulcer formation. Today, moisture is becoming recognised as one of the leading contributory factors in skin ulcer development at the body–seat or body–mattress interface [5,6]. Therefore, the properties of cushions in relation to humidity control is becoming an aspect of greater importance for seat design as well as subjected to greater scrutiny for the purpose of ensuring healthy prolonged sitting.

Initial studies of wheelchair or office seat surface (or cushion) materials were based upon humidity probes [6,7], which have inherent drawbacks including being obtrusive and difficult to accurately place in relation to specific regions of interest. We have previously reported use of discrete, small humidity sensors [8], placed at anatomically relevant locations (under both thighs and ischial tuberosities as well as under the coccyx) to study the relative humidity (RH) changes when sitting on a standard foam cushion. This system allowed us to report similar patterns in RH between thighs and ischial tuberosities. In those experiments [8], the sensors were embedded in small wells in the foam in order to ensure stable positions and record without the subjects’ awareness. However, at the time, we did not fully ascertain the reliability of the sensors or whether some aspect of the RH recorded could have been contributed by non-vapour elements in the experimental procedure (such as pressure). The study presented here reports the results of our more thorough investigation into the reliability, accuracy and effect of additional variables such as loading pressure (simulated sitting) on the output of the chosen sensors. We follow this with results from a preliminary study of humidity sensors to investigate the effect of introducing a gel-pack (standard pressure relieving supplement to wheelchair foam cushions) on the RH profile, including the maximum RH attained and the time to reach maximum RH when compared to a more standard foam cushion.

## 2. Materials and Methods

### 2.1. Data Acquisition Unit

In order to achieve the task of continuously acquiring RH information in real time, a parallel analogue-to-digital converting (ADC) module was considered the most appropriate choice. Based on factors such as reliability and stability, the Pico ADC-11/12 (Pico Technology, Cambridgeshire, UK) was selected as the data sampling unit. The Pico ADC-11/12 has 11 ADC channels with 12-bit resolution and up to 1 kHz sampling frequency per channel. To implement device initialisation, real-time data storage and off-line analysis, a user-friendly graphic user interface was developed with the help of the Visual Basic Integrated Development Environment (IDE; Microsoft Co., Redmond, WA, USA). In addition, the whole measurement system was powered through the computer universal serial bus (USB) port, which increased portability and prevented possible errors when connecting the power supply to the humidity sensors.

### 2.2. Sensor Evaluation

**Figure 1:**
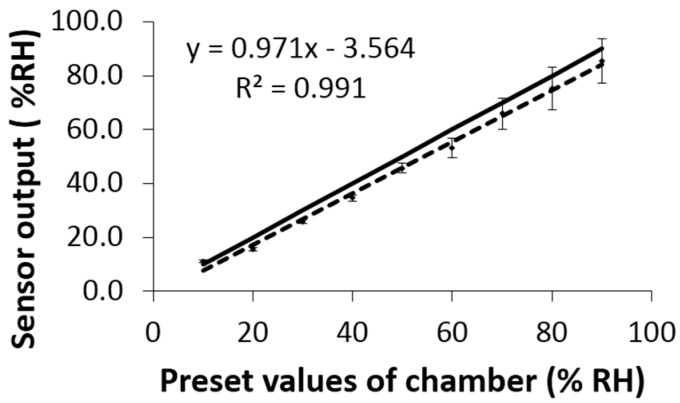
Humidity sensor evaluation with the help of the adjustable standardised humidity chamber using 10% relative humidity (RH) increment each time. Note: the average output of the three humidity sensors [---] (the % RH values were calculated using the manufacturers’ conversion algorithm from the voltage output) approximate linearity corresponding to the traceably calibrated chamber output [―] (chamber temperature was set to 25 °C ± 0.1 °C), suggesting a high reliability and strong correlation. The average values of the measured points are illustrated by diamond markers with the error bars indicating the ±1 standard deviation (SD).

The humidity sensor (HIH4000, Honeywell Co., Morristown, NJ, USA), which is able to convert the moisture information into a corresponding analog voltage signal, was chosen to detect the RH level at the body–seat interface. The advantages of HIH4000 include: (1) simple connection to a microcontroller; (2) easily solderable pins; (3) integrated signal conditioning circuit and (4) multilayer protection construction suitable for hazardous applications. To evaluate the output accuracy of humidity sensors, they were verified over a range of values (10% to 90% RH) using a traceably calibrated humidity chamber (FS990-40V, Design Environmental Ltd.; Ebbw Vale, Gwent, UK, Certificate No. 5493) with 10% RH increment each time. Averaged outcomes of the three sensors for the humidity increasing assessment (Figure 1) showed that deviations between the sensor and the humidity chamber were within the scope of 1% to 5% over the full testing range (10% to 90% RH). This variance further confirmed the suitability of the sensor for our proposed application.

To evaluate the presence and degree of hysteresis for the humidity sensors, a trial using stepped decreases in humidity was also conducted with 10% RH decrement each time: the averaged output of the three sensors also exhibited an approximately linear relationship (*R*^2^ = 0.998) in relation to the preset values of the standardised humidity chamber. In addition, strong correlations were seen between the increasing and decreasing trail results for each sensor (*R*^2^ = 0.997, 0.998 and 0.998, respectively).

### 2.3. Consistency Test under Sand Bag Loading

To simulate the effect of body pressure on the humidity sensors, two 25 kg sandbags were placed on the foam cushion which was embedded with three humidity sensors: one at the rear (similar to the coccyx region of the body) and one on either side of the mid line of the cushion approximately where the middle parts of each thigh would be. An hour-long trial was conducted in a vacant research room with ambient temperature 24.7 °C ± 0.2 °C and 38.6% ± 0.4% RH. To avoid any possible disturbance coming from the change of environmental conditions, the door of the research room was closed during the whole period of the experiment.

**Figure 2:**
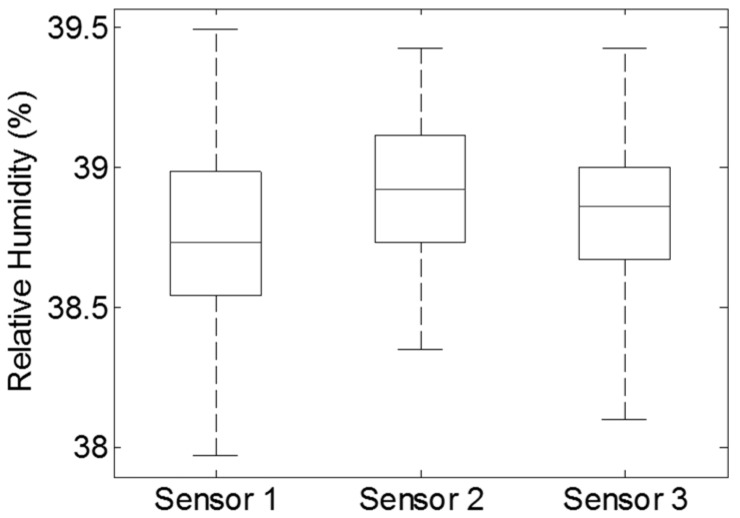
Box and whisker plot of the data from the consistency test for the three humidity sensors using two 25 kg sand bags to simulate the body pressure on the cushion surface [9]. The experiment was conducted in a vacant research room for one hour under the relatively constant ambient conditions (temperature 24.7 °C ± 0.2 °C and 38.6% ± 0.4% RH). Average output RH (±1SD) for the three sensors were: 38.7% (±0.3%), 38.9% (±0.3%) and 38.8% (±0.3%), respectively. Top and bottom whiskers on the figure represent the maximum and minimum values for the corresponding humidity sensors, while the line inside each box indicates the median value. The upper side of each box is the third quartile and the lower side is the first quartile.

Experimental results (Figure 2) indicated that there were no obvious differences (ranging from 38.0% RH to 39.5% RH, *p* > 0.1) between the three humidity sensors under the 50 kg (25 kg × 2 = 50 kg) mass over the period of 1 h. The experiment further attested the measurement consistency of humidity sensors in the same environmental RH. It could be concluded that any difference among the humidity sensors in prolonged sitting experiments could quite confidently be considered as not deriving from the pressure of the mass being applied.

### 2.4. Protocols of Sitting Experiments

#### 2.4.1. Cushions

Two medium density foam wheelchair cushions were used for these experiments with one containing a high-density gel pack placed in a well formed on the uppermost surface (Invacare Ltd., Bridgend, UK). Dimensions of the two cushions were 430 × 484 × 77 mm, except that the gel cushion requires an additional contoured region to accommodate the gel pack. For the purpose of being unobtrusive and imperceptible to subjects, humidity sensors were placed in small recesses (30 mm × 40 mm × 20 mm) cut into the cushion foam (locations of sensors were approximately under the regions of left mid-thigh, right mid-thigh and coccyx relative to the cushions rather than the person sitting on it). There was only one exception for this protocol and that was for the coccyx region of gel pack, where the sensor was placed on the top of it. According to previous studies [7,8], these three locations provide better perspective for monitoring the RH properties of cushions.

Prior to the sitting experiment, both sensor-embedded cushions were covered with the standard black upholstery (Invacare Ltd., Bridgend, UK). After that, they were mounted on identical wheelchairs (Ben 9+: Invacare UK, Bridgend, UK) having a backrest, armrests and adjustable footrests. These additional functions to the wheelchairs created conditions that appeared to generate more consistent sitting postures between subjects [10]. Since upholstery-covered cushions and wheelchair have the same appearance, subjective effects relating to humidity were minimised. As the upholstery is vapour permeable, moisture produced at the body–seat interface is able to penetrate the cover. As a result, the system can effectively collect RH information, though humidity sensors are placed in the cushion underneath the upholstery.

As RH values between the body and the seat would not be expected to vary abruptly over short periods (<1 s), the sampling frequency of the measurement system was set at 1 Hz/sensor. Matlab (MathWorks Co., Natick, MA, USA) and Excel (Microsoft Co., Redmond, WA, USA) were used to process and analyse recorded humidity information.

#### 2.4.2. Participants

Eleven university students (six males and five females) took part in the sitting experiment which had been approved by the Faculty of Health, Sport and Science ethics sub-committee, University of Glamorgan (now University of South Wales). All volunteers who participated in the trial gave written informed consent form prior to the experiment. Age range for the subjects was from 21 to 40 years old, while their body mass index (BMI) was in the range 19.31–26.44 kg·m^−2^. The experimental protocol comprised each subject sitting in their natural (usual) posture for 20 min on either of the wheelchair cushions. Duration of sitting was based on our previous studies [8,10] which found that although the humidity profile initially changed rapidly, it was followed by a relatively ‘stable’ phase after being seated for 15–20 min. Before commencing experiments, subjects were randomly (generated by Microsoft Excel) allocated into one of the two groups, based on whether they were to sit on foam or gel cushion first. Subjects then returned to the research laboratory on the following day at the same time of day to sit on the alternative cushion. The duration between any two tests on the same cushion was at least an hour, allowing sufficient time for the humidity sensors to re-equilibrate with the ambient conditions. At the onset of experiments, the height of footrests for each wheelchair was adjusted to ensure the angles at the knee and hips of the sitting person were approximating 90° and to create an even seating contact across the sitting area. To avoid any impact of garment materials on the outcomes of the sensors, subjects were asked to wear similar cotton pants when attending each experiment. All measurements were conducted in the same research room with monitored ambient temperature and RH (mean ± 1SD during the experimentation period was 21.7 °C ± 0.2 °C and 42.9% ± 2.0% RH, respectively).

#### 2.4.3. Data Smoothing

**Figure 3:**
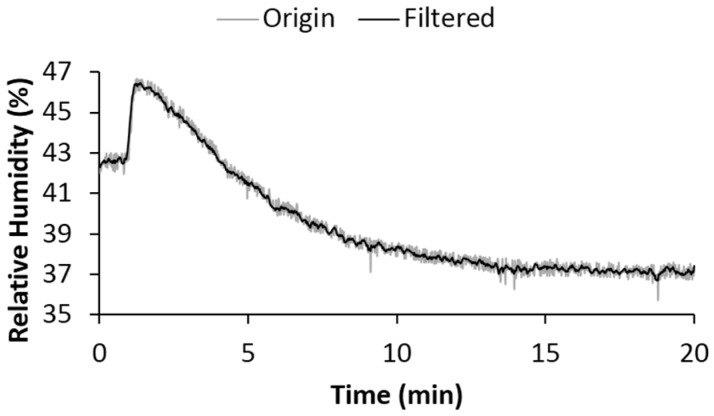
Noise suppression of humidity data with the help of the moving average filter. Original data within the first one minute approximates a straight line (any fluctuations resulting from unwanted noise) because each subject was required to stand in front of their randomly assigned wheelchair and wait for the “start” order before taking a seat. This requirement provided a reference value.

It was necessary to suppress unwanted noisy disturbance combined in the raw data before exploring moisture characteristics of different cushions for prolonged sitting. A moving average filter [11] was used to smooth the data:

(1) $$y_{s}(i)=\frac{1}{2N+1}\left[y(i+N)+y(i+N-1)+\ldots +y(i-N)\right]$$

where $y_{s}(i)$ is the smoothed value for the *i*th data point. For our practical application, N = 3 is the number of neighbouring data points on either side of $y_{s}(i)$, and 2N + 1 represents the filtering span. Comparison between original data and filtered signal was shown in Figure 3, where the spiky noise (unwanted large electronic noise in amplitude) had been affectively removed.

#### 2.4.4. Data Representation

To study the effect of cushion properties on RH, several parameters were employed including average, maximum and median RH values as well as the time to reach the maximum value (*T*_m_) extracted from the 20-min sample data of each participant. Beyond those parameters, data from 30-s epochs at 5th min, 10th min, 15th min and 20th min were used to analyse RH characteristics at different measurement locations.

## 3. Results

A Kolmogorov–Smirnov test was used initially to determine normality of the filtered data. In order to study the relationship among sensors at different measurement positions on each of the two cushion materials, outputs from left mid-thigh, right mid-thigh and coccyx for each individual were examined using one way analysis of variance (ANOVA) and *t*-test. The threshold value for significance was set to 0.05.

### 3.1. Comparison among Different Measurement Locations

For each participant, data from 30-s epochs prior to the end of the time stamps (5 min, 10 min, 15 min and 20 min) were employed to investigate RH features of the three measurement locations. The recorded RH values from each of the three positions were significantly different from each other (ANOVA *p* < 0.01) regardless of time epochs or underlying cushion materials.

**Figure 4:**
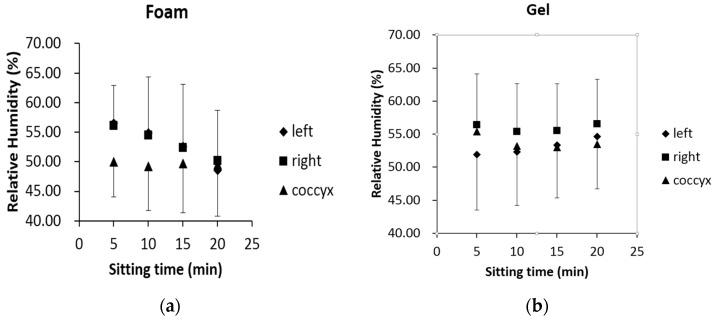
Averaged RH values from the three different measurement locations (left mid-thigh, right mid-thigh and coccyx) using data from 30-s epochs (*n* = 30 × 11 = 330 data points per site) at the time of 5 min, 10 min, 15 min and 20 min. For clarity, the positive error bars represent 1SD of upper values, while negative error bars are the 1SD of the lower values. For the gel cushion, the right mid-thigh produces the largest RH outcomes among the three measured places based on data derived from the 30-s epochs at the 5th min, 10th min, 15th min and 20th min. (**a**) Results of the foam cushion; (**b**) Results of the gel cushion.

It became clear that obvious and consistent differences (paired *t*-test, *p* < 0.01) might exist between the three measurement positions for this small, relatively homogeneous population under these controlled conditions (Figure 4).

### 3.2. Comparison between Different Cushions

The average, maximum and median of RH values (Table 1) show that there exists significant difference between the RH change profile for the foam and the gel wheelchair cushions (*p* < 0.05, paired *t*-test) at the regions of right mid-thigh and coccyx. However, the difference is not significant between the foam and the gel at the area of the left mid-thigh (*p* = 0.12, 0.14, 0.13 for average, maximum and median, respectively, paired *t*-test).

## 4. Discussion

### 4.1. Humidity Sensor Calibration

In the calibration test, all the maximum deviations were within the range given by the datasheet, however, there was no predictable deviation from the preset values of the humidity chamber. For the increasing test, the maximum deviation (5.0% RH) occurred when the preset value of the chamber was 90% RH, whereas, for the decreasing test, the maximum deviation was 6.9% RH when the preset value of the chamber was 80% RH. Though the hysteresis phenomenon between increasing and decreasing tests was not obvious, we would suggest that sufficient time (≥1 h) should be provided to allow humidity sensors to go back to its environmental moisture when undertaking sitting trials where the same seat sees repeated use (as reported here).

**Figure 5:**
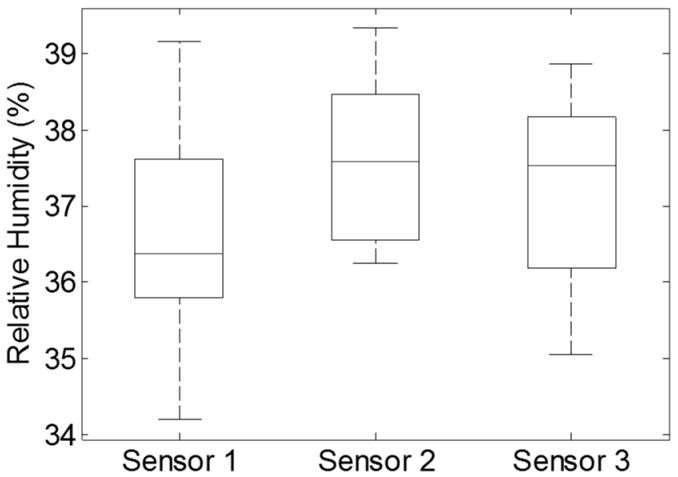
Similarity in output from humidity sensors based on the 30-s epoch data when the standardised humidity chamber was set to 40% RH (similar RH to the sandbag trial in Figure 2).

For the sandbag loading test, the output from the sensors appeared very consistent (*p* > 0.1) with the maximum deviation between the three sensors being 1.5% RH (Figure 2). Data from the 40% RH test in the humidity chamber (similar RH to the sandbag trial) and profiles of humidity measurement (Figure 5) indicated that the humidity sensors were capable of effectively reflecting moisture changes expected and found at the testing locations. Any significant difference among the three sensors during the sitting trials was considered to be associated with cushion properties.

At the current stage, the small size of the variation was deemed to be insignificant in terms of the magnitude changes expected and found in the seating experiments. However, knowing the sensors had individual variations in output gave us the opportunity to visit the minor differences and determine if these were real or related to the inter-sensor differences in the accuracy calibration. Therefore, from the perspective of best practice the authors would respectfully suggest that based on the system performance tests, the calibration data should be integrated into data analysis algorithms in the future when converting voltage outputs to RH values.

### 4.2. RH Distribution over the Interface

No matter which cushion (either foam or gel) is considered, coccyx and right mid-thigh exhibited higher averaged RH values than the left mid-thigh (Table 1). This could be interpreted as the RH not being uniformly distributed over the entire contact surface of the lower body. The best explanation of this lateralisation regarding RH probably lies in all the subjects’ tendency to prefer a better contact with their right side, rather than there being some previously undocumented difference in sweat generation favouring the right lower limb over the left. Handedness was not documented in this experiment; however, this might be a factor worth noting in future experiments as differences in loading due to handedness (sidedness) could introduce an unnecessary confounding variable.

**Figure 6:**
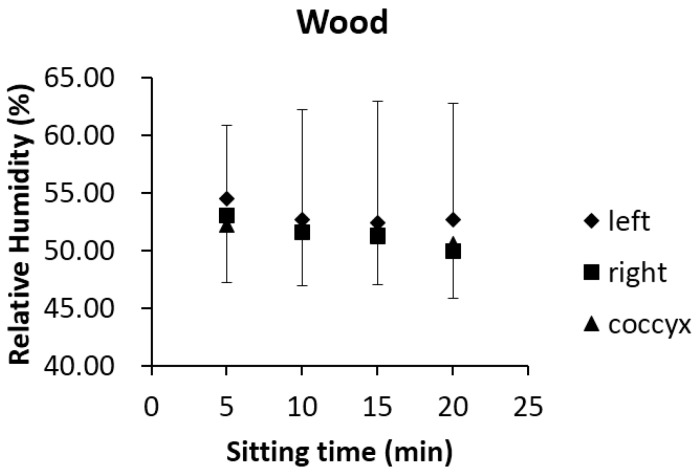
RH output of the wood material using 30-s epoch data at the time of the 5th, 10th, 15th, and 20th min. For clarity, the positive error bars represent 1SD of upper values, while negative error bars are 1SD of lower values.

The foam and gel cushions produced significantly different RH values (average, maximum and median in Table 1) at the three measurement locations (*p* < 0.05). Furthermore, significant differences existed between foam and gel (*p* < 0.05) based on the averaged data over the entire surface. To further investigate the effect of cushion material properties on this result, we conducted an extreme experiment by shaping a chipboard kitchen worktop into a wooden “cushion” equivalent with the same dimensions as the other two cushions (foam and gel). The humidity sensors were embedded in the same positions as the foam and gel cushions, with the unlaminated side facing upward and the rounded edge facing forward and the same upholstery used. In accordance with the experimental protocol, the same subjects (six male and five female) were invited back to sit on the upholstery-covered wooden ‘cushion’, which was mounted on the same wheelchair for 20 min. An interesting finding was that the averaged RH output for the wood ‘cushion’ over the 20 min lay between foam and gel cushions, while the trend in RH was similar to foam: decreasing (Figure 6). These findings indicate that cushion material property appears to influence moisture at the user–seat interface and is a potentially important factor to be considered in the process of seat design.

In addition, all cushions show that the maximal RH can be reached within five minutes (*T*_m_ < 300 s). This finding may be beneficial for cushion manufacturers as less time will be needed to examine those properties of different materials that affect moisture at the user–seat interface [12,13]; which would also be instrumental in reducing time for cushion selection. Consequently, the authors can conceive of the development of a simple, quick and easy system to allow clinicians to offer constructive suggestions regarding suitability of cushion materials in a semi-bespoke manner to wheelchair-reliant patients/end-users or their caregivers [14,15].

### 4.3. Sex Influence on RH

To study the possible impact of different sexes on RH outcomes, recorded data were divided into two groups: male (*n* = 6) and female (*n* = 5). However, no significant difference (*p* > 0.05) was seen between the two groups of data, at any of the three locations for both cushions. This finding further indicated that moisture changes at the contact surface were resulting from aspects of the cushions’ property.

If the accumulated moisture between the body–seat interface were not released through air circulation [16], wetness associated with continuous high pressure could facilitate tissue damage. This relationship has seen growing importance in considering cushion structure in tandem with the cushion materials’ physical properties from the perspective of design on water vapour dissipation [9,12,13]. An example of this is the suggestion that notched seats presumably provide better vertical permeability [7,14].

Patients who use wheelchairs are at greater risk of developing ulcers associated with prolonged ischemia at local areas or due to insufficient ventilation to compressed body parts, especially at the contact surface. Although many ulcer locations are at predictable points on the body, ensuring accurate RH determination at that point may not be possible with the simple approach used here. Owing to the uneven RH distribution over the whole body–seat interface, it will only be possible to provide exact and local descriptions with a grid-like arrays containing many humidity sensors. Therefore, to effectively detect and prevent wetness-caused skin weakening in its early stages, it is necessary to develop a sensor-array-based humidity monitoring system. The results presented here make such a system realisable, as we have shown, the sensors are available and can be used in an unobtrusive manner to gather robust and sufficiently accurate information from the user–seat interface.

### 4.4. Clinical and Physiological Significance

Humidity is one of the most important factors to be considered when selecting an appropriate cushion for people who use wheelchairs. However, there is no universal standard to evaluate the moisture retaining or dispersing properties of different cushions or their covering materials. From the physiological point of view, the mechanism of sweating is constrained when surface pressure is high enough to occlude the blood supply to the sweat gland (reduced blood supply, reduces available interstitial fluid and thus less fluid is available for sweat production). Therefore, there is a push-pull effect between interface pressure, sweat (sensible moisture build up) and humidity. Another factor linked to surface moisture is associated with thermophysiological responses to changes in relative humidity under different thermal environments [17]. Having some understanding of the thermodynamics at the seat cushion interface is essential when selecting wheelchair cushions for wheelchair users in order better understand and ultimately prevent tissue damage due to a prolonged sitting lifestyle where there is a minimal neurological recognition of potential damage.

However, since no material currently offers excellent performance in all aspects of seating comfort (such as interface pressure reduction, efficient thermal flux and fast moisture dissipation), a multi-layer theoretical model has been proposed for clinical applications which divides a cushion into different layers and employs a specific material to that region [18,19]. Our developed system will be beneficial in assessing humidity properties of different materials in situ and eventually could help clinicians give advice to wheelchair users.

## 5. Conclusions

We report the development and testing of a sensor-based measurement system capable of allowing investigation of RH at the user–seat surface interface. The results indicate that the system can reliably monitor RH changes at this important interface and appears unaffected when simulating pressures imposed on sensors during normal sitting.

Compared with previous studies, the system introduced here closely examined the stability and reliability of humidity sensors using traceable calibration methods. These results confirm what we have previously presented with a greater degree of confidence, especially in the stability of the sensors over a period of use, and show if they were affected by the pressure of sitting rather than the humidity alone. Additionally, new data processing methods were employed including that of 30 s epochs statistical analysis and the moving average filter. Furthermore, this paper reports data from a reduced number of sensors and experimental time; reductions being based on our pilot research and confidence in the sensors (sensor number decreased from five to three and trial duration was shortened to 20 min compared with one hour testing time in the preliminary research [8]).

The system also appears capable of discriminating between two commonly used, commercially available cushions and can supply information such as:

1. RH properties at different measurement locations vary (right and left thighs, coccyx). Thus, it is of importance to deploy multiple sensors at the user–seat interface if greater resolution of the RH is needed to determine the highest ‘at risk’ regions. In future research, the optimal number of humidity sensors and their locations will be investigated.
2. RH and changes in RH at the three locations are different for the gel and foam cushion (*p* < 0.05). As cushion composition may have significant impact on moisture at the user–seat interface, it is vital to consider the effects on RH of different materials when selecting cushions for prolonged sitting applications with those in need, such as wheelchair-dependant patients. In addition, this system might help in the future design of materials which can reduce the build-up of moisture at this critical interface zone.
3. For both foam and gel cushions, the time to reach a maximum humidity value during uninterrupted sitting is less than five minutes. This means a shorter testing duration may be possible when evaluating transient humidity properties related to different cushion materials. However, it is important to note that these subjects were not impaired and as a result might have created movements capable of increasing ventilation and reducing interface RH. Therefore, this study should be extended into those populations which are dependent on wheelchairs and may not have the capability of sensing changes at the seat interface.

## Figures and Tables

**Table 1:** Results of RH measurement for foam and gel cushions.

Cushion	Sensor Locations			
		Left Mid-Thigh	Right Mid-Thigh	Coccyx
Foam	Average (% RH)	50.3 ± 5.9	50.6 ± 6.4	51.4 ± 5.6
	Maximum (% RH)	56.0 ± 6.3	53.6 ± 7.0	54.7 ± 5.8
	Median (% RH)	49.6 ± 6.2	50.9 ± 6.6	52.2 ± 5.8
	*T*_m_ (s)	79.6 ± 6.0	119.8 ± 7.1	101.7 ± 4.8
Gel	Average (% RH)	54.0 ± 9.4	56.7 ± 9.2	56.4 ± 8.2
	Maximum (% RH)	59.8 ± 11.0	60.1 ± 9.7	59.9 ± 9.2
	Median (% RH)	53.2 ± 9.1	57.3 ± 9.5	57.2 ± 8.2
	*T*_m_ (s)	115.5 ± 4.4	139.1 ± 9.2	111.8 ± 8.3

Note: Data of humidity sensors from the three locations for each cushion type (*t* = 20 min: each value presented as mean ± 1SD; *n* = 11). Among these parameters, *T*_m_ allows comparison of moisture production rates between the different cushions and measurement sites. The average values can be used to investigate steady-state features of different cushions on RH for prolonged sitting, while the maximum value is a good indicator to assess transient-state characteristics of different cushions on RH.
